# A New Objective Diagnostic Tool for Attention-Deficit Hyper-Activity Disorder (ADHD): Development of the Distractor-Embedded Auditory Continuous Performance Test

**DOI:** 10.3390/jcm13216438

**Published:** 2024-10-27

**Authors:** Ahmet Özaslan, Mehmet Sevri, Elvan İşeri, Barış Karacan, Mehmet Cengiz, Hacer Karacan, Esin Gökçe Sarıpınar, Asiye Uğraş Dikmen, Esra Güney

**Affiliations:** 1 Department of Child and Adolescent Psychiatry, Faculty of Medicine, Gazi University, 06560 Ankara, Turkey; ahmetozaslan@gazi.edu.tr (A.Ö.);; 2 Computer Engineering Department, Faculty of Engineering and Architecture, Recep Tayyip Erdoğan University, 53100 Rize, Turkey; 3 Department of Computer Science, University of Illinois at Chicago, Chicago, IL 60607, USA; 4 Department of Pediatrics, Gülhane Training and Research Hospital, 06010 Ankara, Turkey; 5 Computer Engineering Department, Faculty of Engineering, Gazi University, 06560 Ankara, Turkey; hkaracan@gazi.edu.tr; 6 Department of Public Health, Faculty of Medicine, Gazi University, 06560 Ankara, Turkey

**Keywords:** Attention-Deficit/Hyperactivity Disorder (ADHD), Continuous Performance Test, diagnostic tools, auditory stimuli, distractors, Child Psychiatry

## Abstract

**Background/Objectives**: Attention-Deficit Hyperactivity Disorder (ADHD) is a prevalent neurodevelopmental disorder characterized by inattention, hyperactivity, and impulsivity. Traditional diagnostic methods, which depend on subjective assessments, often lack precision. This study evaluates the validity and reliability of a newly developed diagnostic tool, the Distractor-Embedded Auditory Continuous Performance Test (da-CPT), which integrates auditory stimuli with distractors to enhance the clinical utility of ADHD diagnosis. **Methods**: The study included 160 children aged 6–12 years, comprising 80 with a confirmed ADHD diagnosis and 80 controls. All participants completed the da-CPT, a web-based tool designed to assess inattention, hyperactivity, impulsivity, and timing via an auditory-based task. To validate the da-CPT, participants also completed the Conners’ Parent Rating Scale—Revised Short Form (CPRS-R) and either the MOXO or IVA-2 tests. Data were analyzed using ROC curves and statistical correlations to assess sensitivity, specificity, and overall diagnostic accuracy. **Results**: The da-CPT demonstrated high diagnostic accuracy, with a sensitivity of 91.25% and specificity of 83.75%. ROC analysis indicated that the inattention index had the highest discriminatory power (AUC = 0.881), followed by timing, impulsivity, and hyperactivity (all *p* < 0.01). Furthermore, the da-CPT scores were strongly correlated with ADHD severity (*p* < 0.01). **Conclusions**: This study confirms that the da-CPT is a valid and reliable tool for diagnosing ADHD in children aged 6–12. By incorporating auditory stimuli and distractors, the tool offers a more ecologically valid assessment of ADHD symptoms in clinical settings, improving diagnostic precision and utility.

## 1. Introduction

Attention deficit hyperactivity disorder (ADHD) is a neuropsychiatric disorder that begins in childhood and is characterized by symptoms of inattention, hyperactivity, and impulsivity that are inappropriate for the person’s age [1]. In recent epidemiological studies, the prevalence of ADHD was reported to be 5.9–7.1% in the world [2], while it was shown to be 12.4% in Turkey [3]. ADHD has a considerable and widespread impact, not only on childhood development but also on adulthood, leading to serious consequences. ADHD individuals commonly encounter difficulties in academic achievement, social interactions, and, later, occupational functioning. These challenges can result in various secondary problems, including lowered self-esteem, interpersonal conflicts, and increased susceptibility to substance abuse [4]. The enduring nature of ADHD’s impact on an individual’s life trajectory underscores the critical need for accurate and reliable diagnostic methods. Due to the diverse etiology and behavioral manifestations of ADHD, there is no unique test for diagnosis available. The most widely accepted approach for diagnosing involves utilizing the DSM-5 criteria for clinical evaluations, which usually entails gathering data from various sources, such as parents and teachers, conducting a clinical interview with the child to obtain their perspective, making observations, and utilizing neuropsychological tests [5]. Although traditional approaches are essential, they have limited predictive validity, are based on subjective assessments, and are susceptible to biases from clinicians and informants [6]. Subjectivity in diagnosis may result in inconsistencies and potential risks of overdiagnosis or underdiagnosis, especially for groups like girls [7,8,9]. To address these challenges, it is recommended that incorporating objective assessment tools like continuous performance tests (CPT) in clinical investigations on ADHD could enhance diagnostic sensitivity [10].

The CPT is a computer program characterized by the rapid presentation of a series of visual and/or auditory stimuli (typically numbers, letters, sequences of numbers/letters, or geometric shapes) over a period [11]. Participants are asked to respond when a specific target appears but not to respond to non-targets. While the response to non-target stimuli is called “commission error”, the absence of a response to target stimuli is called “omission error” [12]. Typically, CPT measures selective attention, sustained attention, and impulsivity, which are the main symptoms of ADHD. The participant’s omission errors measure selective attention, commission errors measure impulsivity, and sustained attention is measured by performance during the test, including response time and reaction time variability [5]. CPTs have been used in many studies to distinguish between ADHD and non-ADHD groups, and in most of these studies, it has been shown that the CPT performance of ADHD groups is worse [13,14,15]. Several studies have examined factors influencing CPT performance. These studies have found that performance is influenced by external factors such as time of day, gender, and presence of noise, as well as intrinsic factors like task parameters and distractor inclusion [16,17,18]. For instance, various parameters derived from visual target stimulus-based CPTs are associated with selective attention, impulsivity-hyperactivity, sustained attention, and vigilance. However, Ogundele et al. (2011) state that it is not clear whether CPT’s auditory or visual tasks evaluate the same deficits in ADHD [19]. In a recent study, it was found that inattention was partially independent of the sensory modality, response inhibition (hyperactivity/impulsivity) was modality-specific (visual or auditory target stimulus), and children with ADHD performed lower in the auditory modality [14]. In another study, when discriminant functions were evaluated separately for visual and auditory CPT variables, it was shown that specificity for the auditory task was higher than the visual one. The findings suggest that auditory stimulus is more effective than visual stimulus in differentiating individuals with ADHD from those without the disorder [15]. Therefore, in recent years, interest has shifted from CPTs based on classical visual stimuli to CPTs in which auditory stimuli are the main target. This shift is supported by the idea that auditory stimuli can more accurately simulate attention-demanding tasks in real-life settings, such as classrooms, thus providing greater ecological validity [20,21].

Ecological validity in neuropsychological tests refers to the extent to which the test results accurately reflect real-world outcomes. Individuals with ADHD encounter real-life environments with different distractions, such as visual, auditory, and mixed stimuli. Distractors often impact attention levels. Different distractor characteristics can affect individual performance in cognitive tasks [22]. As with traditional CPTs, neurocognitive tasks used to evaluate patients with ADHD are generally free of distracting stimuli. Therefore, it is assumed that traditional CPTs have low ecological validity, which may explain the weak relationship between CPT performance and behavioral measures measuring attention deficit and hyperactivity [23]. In parallel with this assumption, CPT paradigms containing auditory and visual distractors have been reported to be more reliable and sensitive for the diagnosis of ADHD in children and adolescents than traditional CPTs that do not contain distractors [12].

Contrary to this assumption, it has been suggested recently that individuals with ADHD benefit from distractors and that their cognitive functions can be improved with appropriate stimulation [24]. In a recent study, it was stated that the VR-RVP performance of ADHD and control groups did not differ significantly under conditions with and without distractors [23]. Although distractors are considered to increase ecological validity in ADHD diagnosis, they appear to have a complex effect on CPT performance [25]. Therefore, new studies are needed to develop the most appropriate CPT paradigm using distractors. For example, although it has been suggested in current studies [13,14,15] that auditory target stimulus-based CPTs are more sensitive than visual target stimulus-based CPTs for the diagnosis of ADHD, the effect of distractors on auditory target stimulus-based CPT performance is unknown.

Upon comprehensive evaluation of all the findings, it was determined that there is a necessity to develop an objective tool that assists in diagnosing ADHD while also amending the shortcomings of existing CPTs. To address this requirement within the clinical setting, we have recently developed a novel distractor-embedded auditory continuous performance test (da-CPT). This tool aims to overcome the limitations of existing CPTs by integrating auditory stimuli with a variety of distractors to enhance its ecological validity and clinical utility. The primary aim of this study is to evaluate the validity and reliability of da-CPT in diagnosing ADHD in children aged 6–12 years diagnosed with ADHD through a semi-structured diagnostic interview. The secondary objective is to assess the clinical utility of da-CPT in differentiating between individuals with ADHD and those without the disorder and determine the accuracy of da-CPT’s variable distributions and the proposed diagnostic threshold for ADHD in terms of sensitivity and specificity.

This study represents a significant advancement in the field of ADHD diagnostics, offering a more accurate and reliable approach to clinical assessment through the use of a highly ecologically valid testing paradigm. The main contributions of this study are as follows:

- Development and validation of the da-CPT, a novel tool that integrates auditory stimuli with distractors to enhance ecological validity.
- Demonstration of the da-CPT’s effectiveness in providing a more accurate real-world assessment of ADHD symptoms, particularly in environments with auditory distractions.
- Comparison of the da-CPT’s performance with established tools like MOXO d-CPT [26] and IVA-2 [27], highlighting its high diagnostic accuracy.
- Provision of evidence with high corelation WISC-R test supporting the da-CPT’s clinical utility as a reliable and objective diagnostic tool for ADHD in children.

## 2. Materials and Methods

The da-CPT has a novel approach by incorporating both auditory and visual distractors across its various stages to simulate real-world environments, thus enhancing the ecological validity of the test. This section outlines the participant selection process, the structure of the da-CPT, the technological infrastructure, the scoring method, and the statistical analyses used in the study.

### 2.1. Participants

The study’s participants were recruited from the Child and Adolescent Psychiatry outpatient clinic at Gazi University Faculty of Medicine during the period spanning from November 2019 to June 2022. The study included two groups: an ADHD group and a control group. Participants needed to have an IQ of 85 or higher, as measured by the Wechsler Intelligence Scale for Children-Revised (WISC-R) [28]. This criterion was implemented for both the ADHD and control groups to reduce the influence of cognitive impairment on da-CPT performance. The recruitment of participants with typical cognitive function (IQ≤85) ensured that the da-CPT results specifically indicated ADHD-related attentional deficits, excluding the influence of broader cognitive dysfunction. The ADHD group comprised children between the ages of 6 and 12 who had been diagnosed with ADHD according to the DSM-5 criteria, utilizing the Affective Disorders and Schizophrenia for School-Age Children-Present and Lifetime Version-DSM-5 Turkish Adaptation (K-SADS-PL-DSM-5-T). The exclusion criteria encompassed the presence of comorbid psychiatric disorders, neurological or metabolic diseases, use of any psychoactive drug one month before the application, and having a total IQ score below 85 in the WISC-R test. The control group consisted of children who were of comparable age and sociodemographic features. These children were seeking help at the clinic for non-psychiatric concerns, such as difficulties in family relationships or school adjustment. The inclusion criteria for this study involved individuals who did not have any psychiatric diagnosis after undergoing the K-SADS-PL-DSM-5-T evaluation [29]. The exclusion criteria for this group were the same as those for the ADHD group. Informed consent was obtained from all participants and their parents.

### 2.2. Distractor-Embedded Auditory Continuous Performance Test (da-CPT)

The Distractor-Embedded Auditory Continuous Performance Test (da-CPT) is a novel, web-based assessment tool. The da-CPT evaluates auditory attention and sustaining attention, response inhibition, hyperactivity, and timing, which are the clinical symptoms of ADHD. The present test encompasses 16 min and is composed of eight distinct sections. At the core of da-CPT lies the auditory-based task, wherein participants are required to promptly press the ‘space’ key upon hearing the target auditory stimulus. The meow of the cat, which serves as the target stimulus, is presented at the beginning of the test, and the participant is instructed to press the “space” key on the keyboard only once as soon as the target stimulus is heard. In addition, they are instructed not to respond to non-target stimuli, thereby facilitating the assessment of selective attention and impulsivity. The failure to respond to the target stimulus is regarded as an omission error, which provides insight into the attention profile. Response time variability, which encompasses the duration between the target stimulus and the subsequent response and any fluctuations in response time throughout the testing period, measures the timing profile. The act of responding to a non-target stimulus is classified as a commission error. This type of error is indicative of an impulsivity profile, which is a measure of response inhibition. The analysis of excessive or inappropriate responses, such as multiple vital presses or pressing keys other than the ‘space’ key, is conducted to evaluate the hyperactivity profile. In addition, the test includes both visual (such as images of animals) and auditory distractors (such as ring tones or a baby crying), along with target and non-target stimuli. The addition of distractors serves the purpose of generating real-life situations, thus improving the ecological validity of the test. In this study, the validity and reliability of the da-CPT performance profile in the diagnosis of ADHD were evaluated. The structure of da-CPT, its technological infrastructure, including the rationale behind its design, the types of stimuli used, and its innovative features that distinguish it from other continuous performance tests are detailed in Appendix A.

#### 2.2.1. MOXO Continuous Performance Test (MOXO-CPT)

The MOXO-CPT, developed by Neuro-Tech Solutions Ltd., is a standardized neuropsychological test administered via computer that employs a visual target paradigm [25,26]. The MOXO-CPT distinguishes itself from other CPTs by including additional distractor stimuli designed to simulate real-world distractions. This test has been validated as a reliable and valid tool for assessing ADHD symptoms in children aged 7 to 12 years [26]. In this study, the MOXO-CPT’s performance profile was utilized as a comparative measure to validate the da-CPT’s effectiveness in screening for ADHD symptoms.

#### 2.2.2. Integrated Visual and Auditory Continuous Performance Test, Version 2 (IVA-2)

The IVA-2 is a widely recognized X-type CPT that integrates both auditory and visual stimuli to assess attentional capacities [27]. The IVA-2, which typically takes 15 min to complete, is a valid and reliable tool for aiding in the diagnosis of ADHD and assessing symptom severity in individuals aged 6 years and above. In this study, the IVA-2 served as an additional comparative tool to evaluate the da-CPT’s diagnostic validity and reliability.

#### 2.2.3. Wechsler Intelligence Scale for Children-Revised (WISC-R)

The Wechsler Intelligence Scale for Children-Revised (WISC-R) is a well-established tool for measuring children’s cognitive abilities [28]. It assesses both verbal and performance IQ, providing a comprehensive measure of cognitive functioning. The WISC-R has been validated and standardized in the Turkish population, ensuring its reliability and applicability within this study. The WISC-R was employed to determine the IQ levels of all participants, ensuring that any observed differences in da-CPT performance were not confounded by variations in cognitive ability.

#### 2.2.4. Schedule for Affective Disorders and Schizophrenia for School-Age Children-Present and Lifetime Version-DSM-5-Turkish Version (K-SADS-PL-DSM-5-T)

The K-SADS-PL-DSM-5-T is a semi-structured diagnostic interview designed to evaluate both current and past psychiatric disorders in children and adolescents [29]. This tool integrates information from multiple sources, including the child, parents, and clinical observations, to produce a comprehensive diagnostic assessment. It has been adapted and validated for use in Turkish populations, aligning with the DSM-5 diagnostic criteria. In this study, the K-SADS-PL-DSM-5-T was used to confirm the psychiatric diagnoses of all participants.

#### 2.2.5. Conners’ Parent Rating Scale-Revised Short Form (CPRS-R:S)

The CPRS-R:S is a widely used tool for assessing the severity of ADHD symptoms and monitoring treatment efficacy over time [30]. It consists of 27 items, each rated on a 4-point Likert scale ranging from 0 (not at all) to 3 (very frequently). The scale has been translated into Turkish, and its validity and reliability in the Turkish population have been confirmed. In this study, the CPRS-R:S was utilized to assess the severity of ADHD symptoms and to evaluate the accuracy and consistency of the da-CPT in identifying these symptoms.

### 2.3. Procedure

After the purpose and method of the research were verbally explained, the parents of the participants who agreed to participate in the study were given the CPRS–R: S. Sociodemographic data such as age, gender, and class of both the ADHD group and the control group were recorded on the forms prepared for this study. The ADHD diagnosis was established by an experienced child and adolescent psychiatrist in accordance with DSM-5 criteria, utilizing the K-SADS-PL-DSM-5-T. The same screening procedure was conducted for the control group, and none fulfilled the criteria for ADHD. Moreover, the K-SADS-PL-DSM-5-T were administered to ascertain whether participants fulfilled the diagnostic criteria for psychiatric disorders, which would serve as an exclusion criterion. None of the participants exhibited any psychiatric disorders as delineated in the exclusion criteria based on the K-SADS-PL-DSM-5-T assessment. In the case group, WISC-R was applied to children aged 6–12 years who did not have any additional psychiatric disorders other than ADHD, according to the DSM-5 diagnostic criteria, and after a semi-structured psychiatric interview with the K-SADS-PL-DSM-5-T. In the control group, WISC-R was applied to children who did not have any psychiatric diagnosis after a semi-structured psychiatric interview with DSM-5 diagnostic criteria and the K-SADS-PL- DSM-5-T. After the WISC-R application, da-CPT was applied under expert supervision to the participants who received an IQ score of 85 and above. Participants who underwent da-CPT were randomly administered MOXO CPT or IVA-2 within 3–7 days, at the same time, room, and computer as da-CPT, under the same expert supervision. The ethics committee’s approval of this study was obtained from the Gazi University Faculty of Medicine Ethics Committee (Decision No. 38, 2018).

### 2.4. Statistical Analysis

Statistical analyses were performed to evaluate the diagnostic accuracy and reliability of the da-CPT in identifying ADHD-related symptoms, ensuring the study’s objectives were rigorously met. Data were analyzed using the Statistical Package for Social Sciences (SPSS) version 24.0 and Python. Descriptive statistics, including means, standard deviations, and percentages, were calculated for demographic variables. Sensitivity, specificity, false positive and negative rates, and predictive values of the da-CPT were determined using standard formulas. Additionally, the general accuracy rate and Youden index were calculated to assess the test’s diagnostic value.

Receiver Operating Characteristic (ROC) analysis was used to determine the normative values of the da-CPT, with the area under the ROC curve (AUC) serving as the measure of diagnostic accuracy. Higher AUC values indicate better diagnostic performance, interpreted as follows: 0.90–1.00 (excellent), 0.80–0.90 (good), 0.70–0.80 (moderate), 0.60–0.70 (poor), and 0.50–0.60 (fail) [31]. Independent samples *t*-tests compared da-CPT variable scores between the ADHD and control groups, while Pearson correlation analysis examined relationships between the ADHD index and da-CPT variables. The chi-square test was used to compare qualitative variables, with statistical significance set at a *p*-value of less than 0.05. The power analysis was calculated using the G*Power 3.1.9.7 software. It was determined that at least 64 participants per group would be required to detect a difference with an effect size of 0.5, 80% power, and an alpha error of 0.05. The test development process generally recommends [32] at least 10 participants per step. To further strengthen the study, we included 80 participants in each group (ADHD and control), exceeding the minimum requirement, with similar gender ratios, mean ages, and socioeconomic levels in each group.

## 3. Results

This section presents a comprehensive evaluation of the da-CPT’s effectiveness in diagnosing ADHD in children aged 6–12 years. The analysis includes demographic comparisons between the ADHD and control groups, assessments of diagnostic accuracy through sensitivity and specificity metrics, and ROC curve analyses to evaluate overall performance. Additionally, correlations between da-CPT sub-index scores and ADHD severity, as measured by the Conners’ Parent Rating Scale—Revised Short Form (CPRS-R:S), are examined. Comparative analyses with established tools like the MOXO-CPT and IVA-2 are also included to validate the robustness and reliability of the da-CPT in clinical settings.

### 3.1. Participant Demographics

The demographic characteristics of the participants, including age, gender, and IQ scores, were compared between the ADHD and control groups to ensure no significant differences that could confound the results. As shown in Table 1, statistical analyses, including *t*-tests and chi-square tests, confirmed that the groups were well-matched on these variables, with no significant differences in age or gender distribution, thereby supporting the validity of subsequent cognitive and behavioral comparisons.

### 3.2. Diagnostic Accuracy of da-CPT

The diagnostic accuracy of the da-CPT in identifying ADHD was evaluated by calculating key metrics such as sensitivity, specificity, positive predictive value (PPV), and negative predictive value (NPV). These metrics offer a comprehensive understanding of the test’s ability to correctly identify ADHD cases (true positives) and exclude non-ADHD cases (true negatives). To further validate its effectiveness, the da-CPT’s performance was compared with established tools like the MOXO-CPT and IVA-2. The evaluation was conducted by analyzing key metrics, including sensitivity, specificity, and the Youden Index, which provide insight into each test’s ability to correctly identify ADHD cases and exclude non-ADHD cases. As shown in Table 2, the da-CPT demonstrated high sensitivity and specificity, confirming its reliability as a diagnostic tool for ADHD and highlighting its effectiveness when integrated with other cognitive assessment tools.

**Figure 1:**
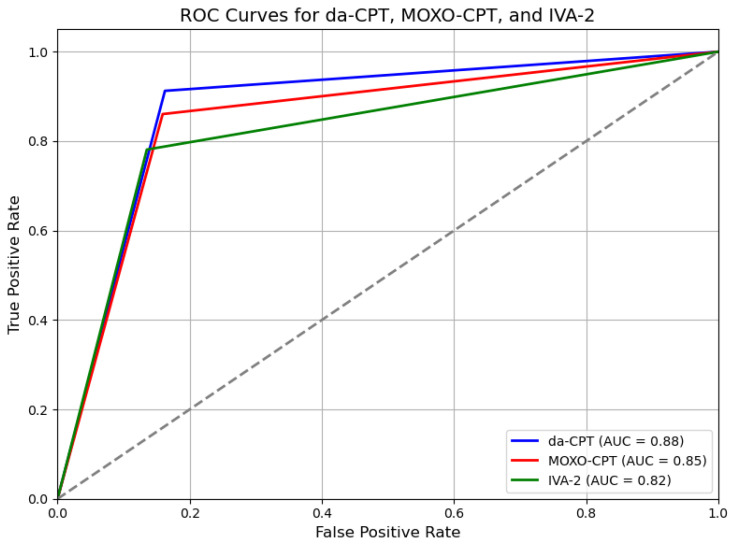
ROC Curves for da-CPT, MOXO-CPT, and IVA-2.

As shown in Table 3, the diagnostic accuracy metrics, including sensitivity, specificity, and AUC values, demonstrate that the da-CPT outperforms the MOXO-CPT and IVA-2 in correctly identifying ADHD cases. The area under the curve (AUC) values indicate the effectiveness of these tools in distinguishing between ADHD and control groups. This is further illustrated in Figure 1, where the ROC curves highlight the higher diagnostic performance of the da-CPT, with a higher AUC, indicating its greater effectiveness in distinguishing between ADHD and control groups.

### 3.3. Correlation Between da-CPT Sub-Indices and ADHD Severity

**Figure 2:**
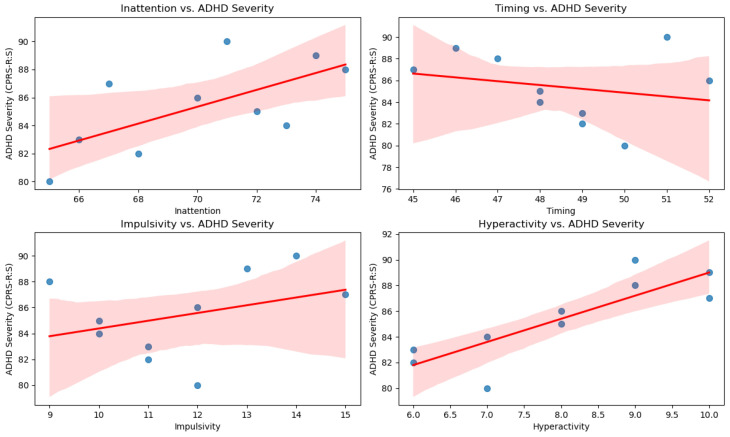
Scatter plots showing the linear relationships between da-CPT sub-index scores (Inattention, Timing, Impulsivity, Hyperactivity) and ADHD severity as measured by CPRS-R:S.

To further assess the clinical utility of the da-CPT, we analyzed the correlations between its sub-indices (inattention, timing, impulsivity, and hyperactivity) and ADHD severity as measured by the Conners’ Parent Rating Scale—Revised Short Form (CPRS-R:S). Table 4 presents the correlation matrix between da-CPT sub-index scores and CPRS-R:S ADHD index scores reveals significant relationships between these variables. ADHD severity, as measured by the CPRS-R:S, was found to be negatively correlated with the inattention and timing sub-indices, and positively correlated with the impulsivity and hyperactivity sub-indices. These findings suggest that higher ADHD severity is associated with lower performance on attention and timing tasks, and greater impulsivity and hyperactivity as measured by the da-CPT. Additionally, Figure 2 visually represents these relationships through scatter plots, highlighting the linear trends between da-CPT scores and CPRS-R:S ratings.

In addition to the correlation analysis, the comparison of da-CPT sub-index scores between the ADHD and control groups also revealed significant differences, as shown in Table 5. The ADHD group had lower mean scores in inattention and timing but higher scores in impulsivity and hyperactivity compared to the control group. between ADHD and non-ADHD populations. These findings underscore the diagnostic utility of the da-CPT in distinguishing between ADHD and non-ADHD populations.

## 4. Discussion

This study evaluated the diagnostic accuracy of the da-CPT for ADHD in children and adolescents aged 6–12 years. We determined the sensitivity, specificity, Youden Index (YI) values, and separate cut-off values for the four subscales. The analysis of these values indicates that the da-CPT has strong discriminant validity, sensitivity, and specificity in diagnosing ADHD in this age group. This highlights its potential as a valuable assistive tool in diagnosing ADHD in children and adolescents.While these results emphasize its potential as a valuable tool in clinical settings for assessing ADHD symptoms, the study should be considered as an initial exploration.

The da-CPT exhibited strong diagnostic accuracy, with a sensitivity of 91.25% (95% CI: 82.80–96.41%) and a specificity of 83.75% (95% CI: 73.82–91.05%). The results were supported by comparing the performance of da-CPT with established tools such as IVA-2 CPT and MOXO CPT, which demonstrated similar levels of sensitivity and specificity. The positive predictive value (PPV) of 84.9%, which exceeds the recommended threshold of 80%, suggests the da-CPT may be useful in combination with other diagnostic measures, including clinical interviews and behavioral rating scales, in providing a more comprehensive assessment of ADHD symptoms. The high PPV of da-CPT suggests that it is a reliable screening tool for providing the diagnosis of ADHD which can be considered an acceptable option in clinical practice [33]. However, it is important to note that, while these results are encouraging, the da-CPT should be regarded as a screening or complementary tool rather than a standalone diagnostic tool.

The da-CPT’s primary innovation lies in its incorporation of both auditory and visual distractors, thereby improving ecological validity and providing a more thorough evaluation of ADHD symptoms in real-world settings. The da-CPT, in contrast to MOXO-CPT, focuses on auditory stimuli, which has been suggested to be more effective in evaluating ADHD-related inattention in practical settings, including classrooms. Furthermore, a significant improvement of the da-CPT over the IVA-2 is its set incorporation of auditory and visual distractors, enhancing its ecological validity and practical relevance in real-world contexts. Another innovation is that the da-CPT incorporates a correction factor for hyperactivity, enabling clinicians to account for excessive key presses, thus enhancing the precision of attention and timing scores. Despite da-CPT’s strengths in assessing auditory based attention, its visual component is less developed than visual stimuli based tools such as MOXO and the IVA-2, which integrates both modalities more evenly. The da-CPT prioritizes auditory stimuli in conjunction with auditory and visual distractors, providing a distinct advantage in evaluating auditory-based attentional control, especially in contexts where auditory tasks are common, an aspect that is often overlooked in other assessment tools.

CPTs are increasingly valued for their objective insights into ADHD symptoms, aiding clinical understanding and parental comprehension of their children’s condition, and alongside clinical evaluations and rating scales, are becoming favored methods for diagnosing and managing ADHD [5,34]. Due to the increasing popularity of using CPTs in the assessment of ADHD, recent systematic reviews primarily examine the psychometric properties of CPTs [35,36,37]. The sensitivity and specificity of commonly utilized CPTs in the diagnosis of ADHD vary widely, ranging from 30% to 90% and 23% to 100%. Our study’s findings align with this range and support the utility of the da-CPT in capturing ADHD, though it is important to contextualize these results within its pilot nature, especially given the lack of comorbidities in our sample. This project developed the first distractor-embedded auditory continuous performance test. Therefore, the sensitivity and selectivity values were compared with those of other frequently employed CPT paradigms. The comparison of its sensitivity and specificity values with other CPT paradigms validates its performance, but further research is needed to fully establish its effectiveness. The obtained results provide a valuable framework for future studies on auditory-focused CPTs that include distractors.

This study utilized ROC analyses to determine the discrimination power of the sub-indexes of da-CPT in distinguishing between children with ADHD and the control group. Discriminative validity is often regarded as acceptable when the Area Under the Curve (AUC) values fall within the range of 0.7 to 0.8, regarded as good when between 0.8 and 0.9, and excellent when beyond 0.9 [31]. Findings indicated that da-CPT’s attention and timing sub-indexes had good discrimination power, while hyperactivity and impulsivity sub-indexes were moderately acceptable. These results are consistent with a recent meta-analysis on ROC analyses of commonly used CPTs [37]. This meta-analysis revealed that the disparities between individuals with ADHD and control groups were more noticeable in terms of omission errors rather than commission errors. In the aforementioned analyses, it is worth mentioning that the AUC values for impulsivity/commission measures in commonly used CPTs were found to be below the acceptable threshold of 0.7 [37].

On the other hand, the sub-indexes of hyperactivity and impulsivity of da-CPT showed higher values, 0.74 and 0.76, respectively, indicating greater discrimination power. Previous research suggests that individuals with ADHD exhibit modality-specific effects (auditory or visual) on commission errors related to impulsivity [14]. Incorporating an auditory target stimulus in the da-CPT paradigm likely enhanced its discriminatory capabilities compared to conventional CPTs. The utilization of both auditory and visual distractors may contribute to the acceptable level of discrimination power exhibited by all subindexes of da-CPT. This is supported by studies indicating that environmental distractors improve the discriminative ability of visual modality CPT [25,38]. The findings of this study emphasize the potential of auditory modalities and the inclusion of distractors in improving the discriminative ability of CPTs for aiding in diagnosing ADHD. However, future studies with larger sample sizes and different age groups are needed to confirm the findings and fully assess the effectiveness of da-CPT for aiding in diagnosing ADHD. This study showed a significant negative correlation between parents’ assessments of ADHD severity and the attention and timing sub-indexes of da-CPT, along with a strong positive correlation in the impulsivity and hyperactivity sub-indexes. The results suggest that da-CPT has considerable promise as a complementary tool for assessing the severity of ADHD symptoms. Furthermore, this could provide helpful details for treatment and planning intervention strategies.

The extent to which CPTs accurately represent the clinical symptom cluster of ADHD remains ambiguous [39]. Previous studies have demonstrated a modest correlation between visual modality CPTs and rating scales completed by parents or teachers [40,41,42]. However, CPTs with an auditory modality exhibit a more substantial alignment, indicating a more reliable concurrence. According to Lehman et al. (2006), children with ADHD exhibited auditory CPT performances more consistent with teacher rating scales, indicating that they were better at detecting attention problems [20]. Another study utilizing an auditory CPT found a strong correlation between CPT performance and scores on the teacher rating scale measuring impulsivity and attention deficit [43]. The findings mentioned above indicate that the modality of CPTs plays a crucial role in reflecting the clinical symptoms of ADHD. Our findings support the growing understanding that auditory-based CPTs more accurately represent ADHD symptoms.

While this study provides promising insights into the use of the da-CPT in ADHD diagnostics, it is important to view these findings as preliminary. Upon analyzing the study’s strengths and its potential impact on clinical practice, the results suggest that the da-CPT could contribute to enhancing our comprehension of ADHD diagnostics, but further research is necessary to confirm its reliability and generalizability. The determination of sensitivity and specificity in typical CPTs typically depends on a threshold that is frequently arbitrary and may not fully distinguish between cases and non-cases [34]. One notable strength of this study lies in its use of ROC analyses to calculate the AUC, which effectively addresses the limitations associated with typical CPTs. The da-CPT is highly adaptable to diverse linguistic and educational backgrounds, making it useful in global contexts, even in resource-limited areas. The simplicity of utilizing da-CPT, which does not require literacy skills, makes it a favorable choice for large-scale studies. The use of universally recognizable stimuli, like cats’ vocalizations, indicates its potential as a global primary care screening tool. This is especially advantageous for mid to low-income countries where limited access to treatment and screening tools is often due to high costs. When looking ahead to potential utilizes da-CPT stands out as a promising screening tool for ADHD in preschool-aged children, especially since current tools are frequently inadequate. Further investigation through larger-scale studies is necessary to explore da-CPT’s practical usefulness and effectiveness, particularly in diverse clinical settings and among ADHD children with comorbidities.

This study has limitations despite providing insights into the utility of da-CPT for ADHD diagnosis. A significant limitation of the study is the small sample size. Furthermore, this study excluded children with psychiatric comorbidities and IQs below 85 to focus on isolating ADHD-specific attentional deficits. While this approach provided a clear view of ADHD-related cognitive performance, it limits the generalizability of the findings to the broader ADHD population, where comorbidities are common. As such, this study represents a preliminary or pilot test of the tool’s efficacy in a more narrowly defined ADHD population. Given the high occurrence of comorbidities in ADHD, particularly those associated with attentional problems [44], future studies need to include a broader range of psychiatric profiles. Additionally, the cross-sectional design of this study limits our understanding of the long-term effectiveness and reliability of da-CPT outcomes. Furthermore, the study’s focus on participants aged 6–12 years may restrict the generalizability of the findings across different ages of ADHD. One limitation of the da-CPT is its inability to replicate real-world distance perception. The tool uses natural sounds and professionally created visual distractors, but the two-dimensional screen and consistently equal volume of sounds might not accurately reproduce different levels of depth and distance experienced by children in their everyday lives. The future versions of the da-CPT and other tools contributing to ecological validity might benefit from including spatial audio and, potentially, three-dimensional visual component. Lastly, technological requirements, like internet access and digital devices, may limit da-CPT’s accessibility in resource-constrained environments, impacting its global utilization. It is essential to acknowledge the limitations to understand this research in the larger context of aiding in ADHD diagnosis and to guide future studies that aim to achieve a more thorough comprehension of the disorder.

## 5. Conclusions

This study has demonstrated that the Distractor-embedded Auditory Continuous Performance Test (da-CPT) is a highly effective complementary diagnostic tool for identifying ADHD in children aged 6–12 years. The da-CPT exhibited strong diagnostic accuracy, with a sensitivity of 91.25% and a specificity of 83.75%. These metrics highlight the tool’s potential reliability and utility in clinical settings. The ROC analyses further underscored the effectiveness of the da-CPT, revealing that the attention and timing sub-indices had particularly high discrimination power, as indicated by their AUC values. The impulsivity and hyperactivity sub-indices also performed well, with AUC values of 0.74 and 0.76, respectively, demonstrating the da-CPT’s ability to differentiate between ADHD and control groups across a broad spectrum of symptoms. Moreover, the significant correlations between the da-CPT sub-indices and ADHD severity suggest that this complementary tool is valuable not only for diagnosis but also for assessing the severity of ADHD symptoms. These findings support the use of da-CPT as a complementary tool for both the identification and management of ADHD. Due to its excellent sensitivity, specificity, and discrimination power, along with the incorporation of innovative technology da-CPT presents itself as an up-and-coming complementary tool in ADHD diagnosis. Moreover, it indicates a significant step towards a more thorough and accurate assessment of attentional processes in children and adolescents. The findings from this research provide a solid basis for future investigations aimed at advancing and improving da-CPT.

## Figures and Tables

**Table 1:** Demographic Characteristics of ADHD and Control Groups.

Characteristic	ADHD Group (n = 80)	Control Group (n = 80)	*p*-Value
**Age (months)**	111.08 ± 21.55	112.06 ± 21.03	0.821 ^1^
**Gender**			0.282 ^2^
Boys (%)	62 (77.5%)	56 (70.0%)	
Girls (%)	18 (22.5%)	24 (30.0%)	

^1^ Independent sample *t*-test, ^2^ Pearson Chi-square test.

**Table 2:** Diagnostic Accuracy Metrics for da-CPT, MOXO-CPT, and IVA-2.

	da-CPT (n = 160)	MOXO (n = 87)	IVA-2 (n = 73)
**Sensitivity**	91.25%	86.05%	86.11%
	(CI: 82.80–96.41%)	(CI: 72.07–94.70%)	(CI: 70.50–95.33%)
**Specificity**	83.75%	84.09%	86.49%
	(CI: 73.82–91.05%)	(CI: 69.93–93.36%)	(CI: 71.23–95.46%)
**Youden Index**	0.75	0.71	0.73

CI: confidence interval.

**Table 3:** ROC Analysis for da-CPT, MOXO-CPT, and IVA-2 in Determining ADHD.

Test	AUC (95% CI)	Cut-Off Scores	*p*-Value	Sensitivity (%)	Specificity (%)
**da-CPT Inattention**	0.881 (0.824–0.938)	72.6	0.001	87.5	83.5
**da-CPT Timing**	0.812 (0.743–0.881)	47.89	0.001	80.0	72.5
**da-CPT Impulsivity**	0.760 (0.685–0.836)	9.84	0.001	72.5	71.2
**da-CPT Hyperactivity**	0.742 (0.662–0.822)	9.50	0.001	70.0	67.2
**MOXO-CPT**	0.850 (0.790–0.910)	50.75	0.002	82.0	78.0
**IVA-2**	0.820 (0.760–0.880)	48.5	0.002	80.0	75.0

**Table 4:** Correlation between da-CPT Sub-Indices and ADHD Severity (CPRS-R:S Scores).

Variables	ADHD Index Score	Inattention	Timing	Impulsivity	Hyperactivity
**ADHD Index Score**	1				
**Inattention**	−0.494 **	1			
**Timing**	−0.474 **	0.805 **	1		
**Impulsivity**	0.285 **	−0.350 **	−0.329 **	1	
**Hyperactivity**	0.271 **	−0.314 **	−0.213 **	0.535 **	1

** *p* < 0.01.

**Table 5:** Comparison of ADHD and Control Groups by scores of sub-index of the da-CPT.

Variable	Groups	N	Mean	SD	t	*p* *
**da-CPT Inattention**	ADHD	80	58.12	14.06	−8.84	<0.001
	Control	80	75.73	10.94		
**da-CPT Timing**	ADHD	80	35.01	13.23	−7.90	<0.001
	Control	80	50.32	11.19		
**da-CPT Impulsivity**	ADHD	80	15.22	8.70	6.21	<0.001
	Control	80	8.57	3.99		
**da-CPT Hyperactivity**	ADHD	80	21.46	17.24	6.10	<0.001
	Control	80	8.53	7.90		

SD: standard deviation, *: Independent sample *t*-test.

**Figure A1:**
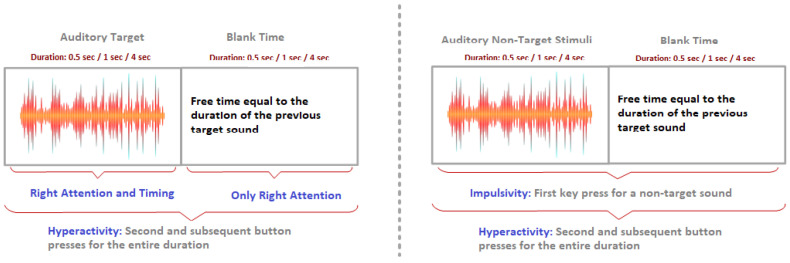
Detection of attention, timing, impulsivity and hyperactivity behaviors.

**Figure A2:**
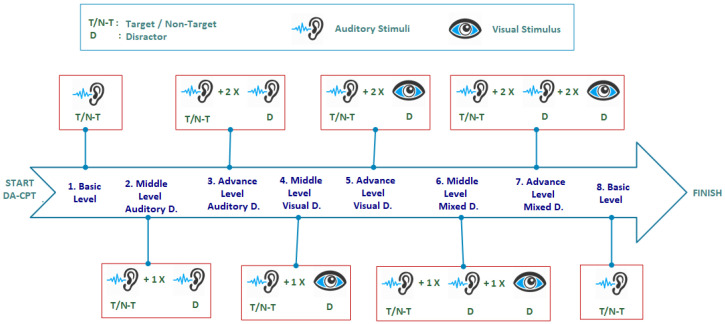
Stages of the Distractor-Embedded Auditory Continuous Performance Test.

## Data Availability

The data supporting the reported results are not publicly available due to patient privacy and confidentiality concerns. Therefore, we are unable to share the data generated or analyzed during this study.

## Abbreviations

The following abbreviations are used in this manuscript:

ADHD	Attention-Deficit/Hyperactivity Disorder
AUC	Area Under the Curve
CI	Confidence Interval
CPRS-R:S	Conners’ Parent Rating Scale—Revised Short Form
CPT	Continuous Performance Test
da-CPT	Distractor-embedded Auditory Continuous Performance Test
DSM-5	Diagnostic and Statistical Manual of Mental Disorders, 5th Edition
K-SADS-PL-DSM-5-T	Schedule for Affective Disorders and Schizophrenia for School-Age Children-Present and Lifetime Version, DSM-5-Turkish Version
MOXO-CPT	MOXO Continuous Performance Test
PPV	Positive Predictive Value
ROC	Receiver Operating Characteristic
SD	Standard Deviation
SPSS	Statistical Package for the Social Sciences
WISC-R	Wechsler Intelligence Scale for Children-Revised
YI	Youden Index

## Appendix A

The (da-CPT is a novel, web-based diagnostic tool specifically designed to evaluate the core clinical symptoms of Attention Deficit Hyperactivity Disorder (ADHD), including inattention, response inhibition, hyperactivity, and timing. By integrating auditory and visual distractions into a controlled testing environment, the da-CPT assesses selective attention and impulsivity through omission errors (failure to respond to the target stimulus) and commission errors (responding to non-target stimuli), respectively. Additionally, it measures timing by tracking response time variability, defined as the interval between the presentation of the target stimulus and the participant’s response, as well as fluctuations in response time throughout the test. To enhance ecological validity and simulate real-world scenarios, the test incorporates a variety of visual (e.g., images of animals) and auditory (e.g., ringing tones, a baby crying) distractors, providing a comprehensive and robust assessment of ADHD symptoms [45,46,47]. The visual distractors used in the ADHD-da-CPT tool were designed by professional cartoon artists, ensuring uniformity in theme and visual style throughout the test. The visual elements, including animal figures, were created using consistent visual fonts and graphic features, specifically focusing on maintaining uniformity in movement capabilities and skeletal structures. This design approach minimizes potential perceptual discrepancies that could otherwise arise from inconsistent visual stimuli.

This section details the da-CPT’s structure, technological infrastructure, including the rationale behind its design, the types of stimuli used, and the innovative features that distinguish it from other continuous performance tests.

### Appendix A.1. Technological Infrastructure

The da-CPT was developed using advanced web technologies to ensure a user-friendly and efficient platform. The frontend, built with HTML5 and Bootstrap, offers a responsive design adaptable to various devices, while PHP and MySQL securely handle data storage and interactions. Real-time data exchange is facilitated by AJAX, supporting smooth test operations and immediate report generation. The interface simplifies patient registration and test scheduling, providing real-time feedback and data visualization for clinical decision-making.

### Appendix A.2. Participant Registration and Demo

The process begins with the registration of the participant by a clinician, such as a child psychologist or psychiatrist. The clinician inputs basic demographic details and assigns a unique identifier to the participant by system. Prior to the main test, the clinician explains the test’s objectives, outlining what the participant should focus on and what to ignore. This session is crucial for ensuring that children aged 6–12 years with ADHD understand the tasks and can engage effectively during the test.

Participants are then introduced to a brief demo session lasting approximately 1.5 min. This demo simulates key elements of the main test, such as the target sound (the sound of a cat meowing) and various non-target and distractor stimuli. The demo explains what the participant should do when hearing the target sound and how to avoid reacting to distractors. Before start the main test screen opens, participants are instructed to “press the spacebar once and only once each time when hear the target sound, while ignoring non-target stimuli!” both verbally and in writing on the application screen. This preparatory phase includes both auditory and visual cues to familiarize the participant with the testing environment. If the clinician deems the participant sufficiently familiar with the test, they may choose to skip this demo.

### Appendix A.3. Main Test Structure

The main test is composed of eight stages, each increasing in complexity and incorporating various distractors. After a countdown timer, the test automatically begins, with no further interaction from the clinician required. The stages are designed to assess different cognitive functions related to ADHD, including attention, timing, impulsivity, and hyperactivity. The test measures several key variables through participant interactions:

- **Correct Attention Responses**: The participant is expected to press the space key on the keyboard as soon as they hear the target sound (e.g., the cat meowing). Correct responses are recorded when the participant presses the key while the target sound is active or immediately afterward in the designated blank interval.
- **Timing**: Timing is assessed by evaluating the precision of the participant’s response, particularly how quickly they react to the target stimulus and maintain consistent reaction times throughout the test. The first key press while the target sound is in progress is used to calculate the timing. Moreover, da-CPT measures response time variability, which includes fluctuations in reaction time between stages. This is a very important measure for the estimation of periods of inattention, under conditions with auditory and visual distractors. In this respect, the test gives a sensitive measure of the lapses in attention so commonly seen in people with ADHD, thus ensuring that periods of inattention and/or delayed responses will be featured in the final outcome of the test.
- **Impulsivity**: Impulsivity is measured by recording instances where the participant presses the key in response to non-target sounds (e.g., the duck quacking or donkey braying).
- **Hyperactivity**: Hyperactivity is calculated based on repeated or inappropriate key presses, particularly when the participant reacts to the same target or non-target stimulus multiple times.

The process of identifying Attention, Timing, Impulsivity and Hyperactivity behaviors is shown in Figure A1.

**Adjusting for Hyperactivity:** A significant innovation in the da-CPT is the correction for hyperactivity’s impact on attention and timing scores. In previous CPT paradigms, hyperactive behavior could lead to inflated scores in attention and timing due to excessive key presses. To address this, the da-CPT incorporates a correction factor that adjusts attention and timing scores if the hyperactivity ratio exceeds two times the sum of attention and timing scores. This correction factor, optimally set at 0.25 times the hyperactivity ratio, ensures that the test results more accurately reflect the participant’s true cognitive abilities. Additionally, the system provides the clinician with the option to modify this correction factor based on individual test results, enhancing the test’s flexibility and accuracy.

**Auditory Target and Non-Target Stimuli:** Target, non-target and distraction sounds consist of professionally recorded natural sounds. The primary target stimulus is the sound of a cat meowing, presented at varying intervals of 0.5, 1, and 4 s. This variability challenges the participant’s ability to sustain attention and respond accurately. In contrast, non-target sounds include other animal noises, such as duck quacking, donkey braying, horse neighing, and wolf howling. These non-target sounds serve to test the participant’s impulsivity by tempting them to react to irrelevant stimuli.

**Auditory and Visual Distractors:** To simulate real-world distractions, the test includes both auditory and visual distractors. Auditory distractors consist of environmental noises like phones ringing, babies crying, car and motorcycle engines, and general crowd sounds. These sounds are strategically placed between the target and non-target stimuli to challenge the participant’s ability to maintain focus amidst distractions. The visual distractors were created by professional designers and graphic designers working in cartoon production as animated cartoons in harmony with the animals with target or distractor sounds. For example, when the cat meowing (target sound) occurs, the screen may display a cat or other animal walking or running. In addition, non-target or distractor sounds might be accompanied by animations of ducks waddling, wolves howling at the moon, or horses galloping across the screen. These animations are designed to be visually engaging, yet challenging, as they test the participant’s ability to prioritize auditory over visual stimuli. This alignment or misalignment between sound and image plays a crucial role in measuring sensory attention, particularly auditory focus, which is a key aspect of ADHD assessment.

### Appendix A.4. Stages and Stimuli

The da-CPT is designed to last 16 min and consists of eight equally timed stages, starting with basic tasks and progressively increasing in complexity, incorporating both auditory and visual distractors. Each stage evaluates different aspects of attention, timing, impulsivity, and hyperactivity by varying the type and number of stimuli presented. This progression allows the da-CPT to systematically assess cognitive functions under increasingly challenging conditions. By carefully combining auditory and visual stimuli, the test accurately measures a participant’s ability to focus and filter out irrelevant information, providing a robust assessment of ADHD-related symptoms. Figure A2 details each stage, including the specific target, non-target, and distractor stimuli used to simulate real-world conditions and enhance diagnostic accuracy.

- **Stage 1:** Target and non-target sounds only—Participants are introduced to the target sound (cat meow) and non-target sounds (e.g., other animal sounds) without any distractors.
- **Stage 2:** Auditory distractors added—Target and non-target sounds are presented along with auditory distractors (e.g., phone ringing, baby crying, crowd noise, motorcycle/car sounds).
- **Stage 3:** Two auditory distractors—Participants hear two auditory distractors simultaneously with target and non-target sounds. For example, combination of a cat meowing (target), a phone ringing (distractor-1) and a baby crying (distractor-2), with the blank screen.
- **Stage 4:** Visual distractors introduced—A visual distractor (e.g., animated animal) is added alongside the target and non-target sounds.
- **Stage 5:** Two visual distractors—Two visual distractors (e.g., cat chasing mouse, eagle flying above a howling wolf) are added, increasing visual complexity.
- **Stage 6:** Mixed auditory and visual distractors—One auditory and one visual distractor are combined with target and non-target sounds.
- **Stage 7:** Multiple auditory and visual distractors—Two auditory and two visual distractors are presented together with target and non-target sounds.
- **Stage 8:** Return to basic level—Only target and non-target sounds are presented again without any distractors, to assess performance consistency.

### Appendix A.5. Reporting and Analysis

Upon completion of the test, the system generates a detailed report that is accessible through a clinician interface. This report includes both raw scores and adjusted scores for attention, timing, impulsivity, and hyperactivity, presented in graphical and tabular formats. The clinician can review these results in the context of the participant’s performance, allowing for a more informed diagnostic process. The da-CPT’s architecture is distinguished by its emphasis on ecological validity and its innovative approach to handling hyperactivity. By simulating real-world distractions and correcting for hyperactivity’s impact on other cognitive measures, the da-CPT provides a more accurate and reliable assessment tool for diagnosing ADHD in children aged 6–12 years. Furthermore, the strategic integration of both congruent and incongruent visual and auditory stimuli enhances the test’s ability to differentiate between visual and sensory attention, offering a comprehensive evaluation of the participant’s cognitive functions.

### Appendix A.6. Public Demo Access for Researchers and the Public

To promote transparency and encourage broader research use, we have developed an open-access demo platform for the da-CPT. The demo can be accessed and used freely for research purposes only and cannot be used for commercial purposes through the following link: https://mehmetsevri.github.io/nadacpt.html (accessed on 23 October 2024).

## References

[1] American Psychiatric Association (2022). Neurodevelopmental disorders. Diagnostic and Statistical Manual of Mental Disorders.

[2] Willcutt E.G. (2012). The prevalence of DSM-IV attention-deficit/hyperactivity disorder: A meta-analytic review. Neurotherapeutics.

[3] Ercan E.S., Polanczyk G., Akyol Ardıc U., Yuce D., Karacetin G., Tufan A.E., Tekden M. (2019). The prevalence of childhood psychopathology in Turkey: A cross-sectional multicenter nationwide study (EPICPAT-T). Nord. J. Psychiatry.

[4] MacDonald L., Sadek J. (2023). Management Strategies for Borderline Personality Disorder and Bipolar Disorder Comorbidities in Adults with ADHD: A Narrative Review. Brain Sci..

[5] Hall C.L., Valentine A.Z., Groom M.J., Walker G.M., Sayal K., Daley D., Hollis C. (2016). The clinical utility of the continuous performance test and objective measures of activity for diagnosing and monitoring ADHD in children: A systematic review. Eur. Child Adolesc. Psychiatry.

[6] Lange K.W., Hauser J., Lange K.M., Makulska-Gertruda E., Takano T., Takeuchi Y., Tucha O. (2014). Utility of cognitive neuropsychological assessment in attention-deficit/hyperactivity disorder. ADHD Atten. Deficit Hyperact. Disord..

[7] Serra-Pinheiro M.A., Mattos P., Angélica Regalla M. (2008). Inattention, hyperactivity, and oppositional–defiant symptoms in Brazilian adolescents: Gender prevalence and agreement between teachers and parents in a non–English speaking population. J. Atten. Disord..

[8] Ginsberg Y., Quintero J., Anand E., Casillas M., Upadhyaya H.P. (2014). Underdiagnosis of attention-deficit/hyperactivity disorder in adult patients: A review of the literature. Prim. Care Companion CNS Disord..

[9] Quinn P.O., Madhoo M. (2014). A review of attention-deficit/hyperactivity disorder in women and girls: Uncovering this hidden diagnosis. Prim. Care Companion CNS Disord..

[10] Stokes J.D., Rizzo A., Geng J.J., Schweitzer J.B. (2022). Measuring Attentional Distraction in Children with ADHD Using Virtual Reality Technology with Eye-Tracking. Front. Virtual Real..

[11] Riccio C.A., Reynolds C.R. (2001). Continuous performance tests are sensitive to ADHD in adults but lack specificity. A review and critique for differential diagnosis. Ann. New York Acad. Sci..

[12] Berger I., Slobodin O., Cassuto H. (2017). Usefulness and Validity of Continuous Performance Tests in the Diagnosis of Attention-Deficit Hyperactivity Disorder Children. Arch. Clin. Neuropsychol..

[13] Roebuck H., Freigang C., Barry J.G. (2016). Continuous Performance Tasks: Not Just About Sustaining Attention. J. Speech Lang. Hear. Res..

[14] Schmidt S.L., Simões E.D.N., Novais Carvalho A.L. (2019). Association Between Auditory and Visual Continuous Performance Tests in Students with ADHD. J. Atten. Disord..

[15] Simões E.N., Carvalho A.L.N., Schmidt S.L. (2021). The Role of Visual and Auditory Stimuli in Continuous Performance Tests: Differential Effects on Children with ADHD. J. Atten. Disord..

[16] Corkum P.V., Siegel L.S. (1993). Is the Continuous Performance Task a valuable research tool for use with children with Attention-Deficit-Hyperactivity Disorder?. J. Child Psychol. Psychiatry.

[17] Ballard J.C. (1996). Computerized assessment of sustained attention: A review of factors affecting vigilance performance. J. Clin. Exp. Neuropsychol..

[18] Riccio C.A., Reynolds C.R., Lowe P., Moore J.J. (2002). The continuous performance test: A window on the neural substrates for attention?. Arch. Clin. Neuropsychol..

[19] Ogundele M.O., Ayyash H.F., Banerjee S. (2011). Role of computerised continuous performance task tests in ADHD. Prog. Neurol. Psychiatry.

[20] Lehman E.B., Olson V.A., Aquilino S.A., Hall L.C. (2006). Auditory and visual continuous performance tests: Relationships with age, gender, cognitive functioning, and classroom behavior. J. Psychoeduc. Assess..

[21] Shapiro S.K., Herod L.A. (2013). Tasks in the Assessment of Attention-Deficit Hyperactivity. Disruptive Behavior Disorders in Childhood.

[22] Pelham W.E., Waschbusch D.A., Hoza B., Gnagy E.M., Greiner A.R., Sams S.E., Vallano G., Majumdar A., Carter R.L. (2011). Music and video as distractors for boys with ADHD in the classroom: Comparison with controls, individual differences, and medication effects. J. Abnorm. Child Psychol..

[23] Hong N., Kim J.J., Kwon J.H., Eom H., Kim E. (2022). Effect of Distractors on Sustained Attention and Hyperactivity in Youth with Attention Deficit Hyperactivity Disorder Using a Mobile Virtual Reality School Program. J. Atten. Disord..

[24] Baijot S., Slama H., Söderlund G., Dan B., Deltenre P., Colin C., Deconinck N. (2016). Neuropsychological and neurophysiological benefits from white noise in children with and without ADHD. Behav. Brain Funct..

[25] Cassuto H., Ben-Simon A., Berger I. (2013). Using environmental distractors in the diagnosis of ADHD. Front. Hum. Neurosci..

[26] Berger I., Goldzweig G. (2010). Objective measures of attention-deficit/hyperactivity disorder: A pilot study. Isr. Med. Assoc. J..

[27] Moreno-García I., Delgado-Pardo G., Roldán-Blasco C. (2015). Attention and response control in ADHD. Evaluation through integrated visual and auditory continuous performance test. Span. J. Psychol..

[28] Savaşır I., Şahin N. (1995). Wechsler Çocuklar Için Zeka Ölçeği (WISC-R) el Kitabı.

[29] Unal F., Oktem F., Cetin Cuhadaroglu F., Cengel Kultur S.E., Akdemir D., Foto Ozdemir D., Cak H.T., Unal D., Tiras K., Aslan C. (2019). Reliability and Validity of the Schedule for Affective Disorders and Schizophrenia for School-Age Children-Present and Lifetime Version, DSM-5 November 2016-Turkish Adaptation (K-SADS-PL-DSM-5-T). Turk. J. Psychiatry.

[30] Kaner S., Buyukozturk S., Iseri E. (2013). Conners parent rating scale-revised short: Turkish standardization study/Conners anababa dereceleme olcegi-yenilenmis kisa: Turkiye stardardizasyon calismasi. Arch. Neuropsychiatry.

[31] Safari S., Baratloo A., Elfil M., Negida A. (2016). Evidence based emergency medicine; part 5 receiver operating curve and area under the curve. Emergency.

[32] Wu D., Akl E.A., Guyatt G.H., Scott I., Oderda G.M. (2014). Methodological Survey of Designed Uneven Randomization Trials (DU-RANDOM): A Protocol. Trials.

[33] Cortese S., Solmi M., Michelini G., Bellato A., Blanner C., Canozzi A., Eudave L., Farhat L.C., Højlund M., Köhler-Forsberg O. (2023). Candidate diagnostic biomarkers for neurodevelopmental disorders in children and adolescents: A systematic review. World Psychiatry.

[34] Bellato A., Hall C.L., Groom M.J., Simonoff E., Thapar A., Hollis C., Cortese S. (2023). Practitioner Review: Clinical utility of the QbTest for the assessment and diagnosis of attention-deficit/hyperactivity disorder—A systematic review and meta-analysis. J. Child Psychol. Psychiatry.

[35] Huang-Pollock C.L., Karalunas S.L., Tam H., Moore A.N. (2012). Evaluating vigilance deficits in ADHD: A meta-analysis of CPT performance. J. Abnorm. Psychol..

[36] Tallberg P., Råstam M., Wenhov L., Eliasson G., Gustafsson P. (2019). Incremental clinical utility of continuous performance tests in childhood ADHD—An evidence-based assessment approach. Scand. J. Psychol..

[37] Arrondo G., Mulraney M., Iturmendi-Sabater I., Musullulu H., Gambra L., Niculcea T., Banaschewski T., Simonoff E., Döpfner M., Hinshaw S.P. (2023). Systematic Review and Meta-analysis: Clinical Utility of Continuous Performance Tests for the Identification of Attention-Deficit/Hyperactivity Disorder. J. Am. Acad. Child Adolesc. Psychiatry.

[38] Berger I., Cassuto H. (2014). The effect of environmental distractors incorporation into a CPT on sustained attention and ADHD diagnosis among adolescents. J. Neurosci. Methods.

[39] De Rossi P., Pretelli I., Menghini D., D’Aiello B., Di Vara S., Vicari S. (2022). Gender-Related Clinical Characteristics in Children and Adolescents with ADHD. J. Clin. Med..

[40] Forbes G.B. (1998). Clinical utility of the Test of Variables of Attention (TOVA) in the diagnosis of attention-deficit/hyperactivity disorder. J. Clin. Psychol..

[41] McGee R.A., Clark S.E., Symons D.K. (2000). Does the Conners’ Continuous Performance Test aid in ADHD diagnosis?. J. Abnorm. Child Psychol..

[42] Kim J., Lee Y., Han D., Min K., Kim D., Lee C. (2015). The utility of quantitative electroencephalography and Integrated Visual and Auditory Continuous Performance Test as auxiliary tools for the Attention Deficit Hyperactivity Disorder diagnosis. Clin. Neurophysiol..

[43] Lasee M.J., Choi H. (2013). Evidence of Reliability and Validity for a Children’s Auditory Continuous Performance Test. SAGE Open.

[44] Özaslan A., İşeri E., Erermiş H.S. (2019). Özel Öğrenme Bozukluklarında Prognoz, Prognostik Faktörler ve Erken Tedavinin Etkisi. Çocuk ve Gençlik Çağı Ruhsal Hastalıklarında İzlem ve Süreç: Prognostik Faktörler ve Erken Tedavi Yaklaşımlarının Etkisi.

[45] Romero-Ayuso D., del Pino-González A., Torres-Jiménez A., Juan-González J., Celdrán F.J., Franchella M.C., González P. (2024). Enhancing Ecological Validity: Virtual Reality Assessment of Executive Functioning in Children and Adolescents with ADHD. Children.

[46] Tyburski E., Mak M., Sokołowski A., Starkowska A., Karabanowicz E., Kerestey M., Jansari A.S. (2021). Executive Dysfunctions in Schizophrenia: A Critical Review of Traditional, Ecological, and Virtual Reality Assessments. J. Clin. Med..

[47] Elbaum T., Braw Y., Lev A., Rassovsky Y. (2020). Attention-Deficit/Hyperactivity Disorder (ADHD): Integrating the MOXO-dCPT with an Eye Tracker Enhances Diagnostic Precision. Sensors.

